# Canine Epidermal Keratinocytes (CPEK) Grown in Monolayer Are Not Representative of Normal Canine Keratinocytes for Permeability Studies: Pilot Studies

**DOI:** 10.3390/vetsci9010025

**Published:** 2022-01-11

**Authors:** Rosanna Marsella, Rachel Wilkes, Kim Ahrens

**Affiliations:** Department of Small Animal Clinical Sciences, College of Veterinary Medicine, University of Florida, Gainesville, FL 32610, USA; rachelsusansanford@gmail.com (R.W.); sciencematters2me@gmail.com (K.A.)

**Keywords:** transepithelial electric resistance (TEER), canine epidermal keratinocytes, tight junction

## Abstract

Canine progenitor epidermal keratinocytes (CPEK) are used as canine keratinocyte cell line. Their suitability for skin barrier studies is unknown. Measurement of transepithelial electric resistance (TEER) evaluates epithelial permeability. We compared TEER and tight junction (TJ) expression in CPEKs and normal keratinocytes (NK) harvested from biopsies of normal dogs. CPEKs and NK were grown until confluence (D0) and for 13 additional days. Slides were fixed on D0 and stained with ZO-1 and claudin-1 antibodies. Five images/antibody were taken, randomized and evaluated blindly by three investigators for intensity, staining location, granularity, and continuousness. Cell size and variability were evaluated. TEER increased overtime to 2000 Ohms/cm in NK, while remained around 100–150 Ohms/cm in CPEK. ANOVA showed significant effect of time (*p* < 0.0001), group (*p* < 0.0001) and group x time interaction (*p* < 0.0001) for TEER. Size of CPEKs was significantly (*p* < 0.0001) smaller and less variable (*p* = 0.0078) than NK. Intensity of claudin-1 staining was greater in CPEKs (*p* < 0.0001) while granularity was less in CPEKs (*p* = 0.0012). For ZO-1, cytoplasmic staining was greater in CPEK (*p* < 0.0001) while membrane continuousness of staining was greater in NK (*p* = 0.0002). We conclude that CPEKs grown in monolayer are not representative of NK for permeability studies.

## 1. Introduction

Keratinocytes are increasingly recognized as playing an important role in orchestrating the immune response and much attention has been devoted in recent years on the importance of skin barrier function in dermatological diseases like atopic dermatitis [1]. In veterinary medicine it has been customary to use canine progenitor epidermal keratinocyte (CPEKs) for in vitro studies and consider them as a substitute for canine keratinocytes [2,3]. This is done for convenience as CPEKs can be purchased and no biopsies from dogs are needed. CPEKs grow readily and many publications have been done using CPEKs as representative of what normal keratinocytes (NK) would do [2,3]. The alternative approach would be to take biopsies from dogs, harvest keratinocytes and establish primary cultures [4]. This approach is more labor intensive and the need for skin biopsies creates some practical limitations.

A reason to use cell cultures is the evaluation of keratinocytes responses without the influence of dermal inflammation. This is particularly helpful in studies on atopic dermatitis as there is controversy on whether skin barrier dysfunction in canine atopic dermatitis is primary or secondary to inflammation [5,6]. Increased transepidermal water loss has been reported in atopic dogs [7,8] and skin barrier impairment has been linked to increased allergic sensitization [9]. Tight junctions (TJ) play an important role in modulating permeability of epithelia [10,11] and recent studies have described a decreased TJ expression in biopsies of atopic dogs compared to normals [12,13]. A recent publication described that CPEK 3D epidermal equivalent have staining patterns of TJ proteins such as Zonula Occludens-1 (ZO-1) and claudin-1 with some similarities of normal canine skin [14].

Establishment of a cell culture and measurement of transepithelial electrical resistance (TEER) is a widely used in vitro approach to measure the strength of connection between epithelial cells and the permeability of the epithelia [15,16]. With this methodology the higher the TEER, the stronger is the connection established between epithelial cells and the lower is the permeability of the epithelial barrier [17,18].

The purpose of our study was twofold. First was to compare TEER of CPEKs with TEER of NK grown in monolayer to see if CPEKs can be used as substitute for normal canine keratinocytes in studies on skin barrier permeability. The second aim was to compare the morphology of CPEKs with the one of NK and compare ZO-1 and claudin-1 protein expression between the two populations of cells.

## 2. Materials and Methods

All procedures involving research animals in this study had been reviewed and approved by Institutional Animal Care and Use committee.

### 2.1. Animals and Skin Biopsy Collection

Normal, healthy research beagles (*n* = 4) were used to harvest skin biopsies. Two 8-mm biopsy punch biopsies were collected from the inguinal area of each dog. The skin was cleaned with ethanol and betadine before taking the biopsy. Sodium bicarbonate and lidocaine was injected before biopsy harvesting. The site was sutured routinely. The biopsy was placed in sterile PBS on ice, then washed with betadine. Each biopsy was cut in half then placed in 1.25 U/l dispase overnight. The epidermis was removed using sterile forceps and floated on TrypLE (Gibco 12563-011) for 30 min.

### 2.2. Keratinocyte Culture

Keratinocytes were harvested by agitating the epidermis, then cultured using CellnTec (CnT-09) media. Cells were cultured on T25 flasks until approximately 60% confluent. These cells were trypsinzied using TrypLE and re-seeded on a new flask and let grow for overnight. The media was changed and all cells which were not attached were thrown away with the media. The cells were grown until approximately 60% confluent. This process was repeated. This process only allows for basal keratinocytes to proliferate in culture, as other cell types have a much slower growth process and will be rapidly overgrown by keratinocytes [19]. The exception to this is fibroblasts. If there was any fibroblast contamination in the keratinocytes, differential trypsinization was performed to detach the fibroblasts and not keratinocytes. After proliferation these were frozen in cell culture freezing media (Gibco 12638-010) overnight at −80 °C then kept in liquid nitrogen for storage. Cells were seeded onto Lab-TeK II Chamber Slides (Thermo Fisher 154526, Waltham, MA, USA) at 1 × 10^5^ cells per well. Once cells reached confluency, this time point was labeled as Day 0 (D0). CPEK cells were purchased from CellnTec (CPEK). These cells were grown with CellnTec (CnT-09) media. They were seeded at the same concentrations as the primary keratinocytes.

### 2.3. TransEpithelial Electrical Resistance (TEER) Measurement

To measure the transepithelial electrical resistance (TEER) 1.875 × 10^4^ cells per well were grown on transwell inserts (costar 3470) in 24 well plates. We used *n* = 4 normal dogs. Duplicate wells were seeded for each dog and media was changed every other day. Six CPEK wells were seeded. Each pair of CPEK wells was treated as one ‘dog’ effectively having three CPEK subjects, with duplicate wells. We used 250 µL CellnTec (CnT-09) media in the well and 500 µL media in the plate below the well. We also seeded 1.1 × 105 cells in wells without inserts, to be able to observe confluency. One well with the transwell insert was made with only media and no keratinocytes as a ‘blank’ reading. This well’s media was changed when the cells media was changed. Once confluent readings were taking using the EVOM2 Epithelial Voltohmmeter by World Precision Instruments. The readings of the duplicate wells were averaged. The blank well reading was taken every day and subtracted from the averaged readings. The instrument was rinsed in media between each dog and placed in 70% ethanol after all reading completed each day. TEER was measured daily up to 13 days after confluence. We followed the protocol of our colleagues in human dermatology who have perfected this method [20].

### 2.4. Immunofluorescence Staining

**Figure 1:**
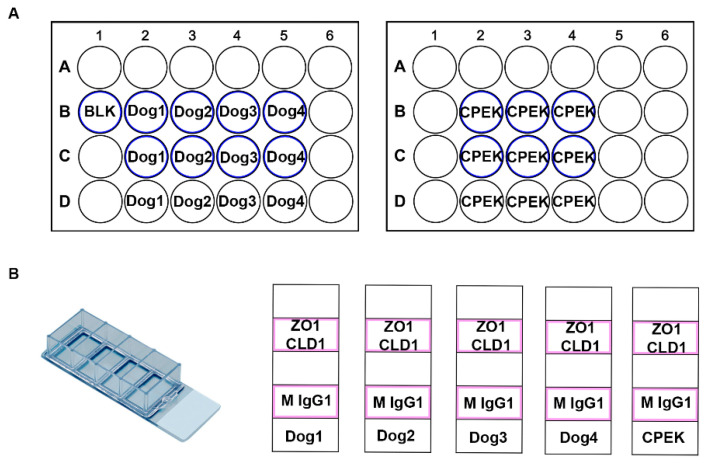
These are diagrams of our cell culture set up. (**A**) Represents the TransEpithelial Electrical Resistance (TEER) well set up. We had two 24 well plates with transwell inserts. The wells which had transwell inserts are represented with a blue outline. (**B**) Represents the chamber slide set up. These were Lab-TeK II Chamber Slides with 4 wells. Cells were grown in the chambers outlined in pink. ZO-1 (Thermo Fisher 339100) and Claudin-1 (Abcam ab15098) were used as primary antibodies with a secondary stain of donkey anti mouse IgG 488 (Thermo Fisher A10042) and donkey anti rabbit 594 (Thermo Fisher A21202). Mouse IgG1 Isotype Control (Thermo Fisher MA5-14453) was used as a negative control.

Four normal dogs were used (*n* = 4) for this experiment (Figure 1). Each dog’s cells were seeded on a 4 chamber Lab-TeK II Chamber Slide (Thermo Fisher 154526) at 1 × 10^5^ cells per well. CPEK cells (*n* = 1) were also seeded onto one four-chamber slide. Each chamber of these slide was dual stained with two primary antibodies and two secondary antibodies of different colors, described below. For a negative control, mouse IgG1 Isotype Control antibody was stained for each dog or CPEK.

Cells were fixed using 3.7% PFA on day of confluence then rinsed in PBS and diH_2_O. Slides were dried overnight at RT and 20 min at 60 C. All dilutions for immunofluorescence were diluted with common antibody diluent (Biogenex HK156-5K, Fremont, CA, USA). Antigens were retrieved by placing slides in Tris-EDTA pH 9 buffer for 15 min in a rice cooker (Aroma ARC-150SB) on ‘steam’, then cooled in rice cooker for 10 min and washed with PBS. The slides were incubated with Power Block (Biogenex HK083-50K) for 10 min, power block was tapped off without being rinsed and blocked again using normal donkey serum (Jackson ImmunoResearch 017-000-121, West Grove, PA, USA) diluted to 10% for 20 min. Slides were washed with PBS and incubated with primary antibody overnight at 4 °C. Slides were washed with PBS. Secondary donkey anti mouse IgG 488 (Thermo Fisher A10042) and donkey anti rabbit 594 (Thermo Fisher A21202), diluted 1:500, was added and incubated for 45 min in the dark RT. Slides were washed with PBS, coverslipped with VECTASHIELD Antifade Mounting Medium with DAPI (H-1200), and sealed using nail polish. Antibodies used were: ZO-1 (Thermo Fisher 339100) 1:150; Claudin-1 (Abcam ab15098) 1:20; Mouse IgG1 Isotype Control (Thermo Fisher MA5-14453) 1:150. The ZO-1 human sequence (NCBI Reference Sequence: NP_003248.3) has 93% sequence homology to the canine ZO-1 sequence (NCBI Reference Sequence: NP_001003140.1) and the antibody has been validated by Thermo Fisher to react with the canine sequence. The Claudin-1 human sequence (NCBI Reference Sequence: NP_066924.1) has 93% sequence homology to the canine Claudin-1 sequence (NCBI Reference Sequence: XP_850248.1). The antibodies used in this study were selected based on prior published work done by our lab on skin biopsies of normal and atopic dogs [13].

### 2.5. Evaluation Staining and Cell Morphology

Fluorescent pictures were taken at 20× on an EVOS fluorescence microscope. Intensity and shutter speed were determined by testing settings on both atopic and normal chambers. The same settings were used for each antibody. Five pictures of each antibody for each chamber of the four-chamber slide were taken to get a good representation of all cells in the chamber. Each image was taken viewing only one color. Images for each antibody were taken, randomized, and evaluated blindly by three investigators unaware of sample source for the images. Observers scored staining for intensity (scale from 0 to 5, with higher numbers indicating stronger intensity), location (nuclear, cytoplasmic, or membrane), granularity and continuousness of staining. Cells were evaluated for size (scale from 1 to 3, with 1 being small, 2 being medium and 3 being large), and uniformity of size. The score for each picture was averaged if the variable was an integer. If the variable was categorical the percent of observers who indicated each category was calculated for each picture. These scores were averaged for each chamber, where each chamber was one dog’s score for one antibody.

### 2.6. Statistical Analysis

Due to the difference between variability in normal dogs (higher variability) and CPEK cells (lower variability) we used the Welch’s unequal variances *t*-test for statistical analysis of immunofluorescence data. Two-way repeated measures ANOVA and Sidak’s multiple comparisons test were used for TEER comparisons. P less than 0.05 was considered significant. Statistics were completed using Graph Pad Prism 8.

## 3. Results

### 3.1. TransEpithelial Electrical Resistance (TEER)

**Figure 2:**
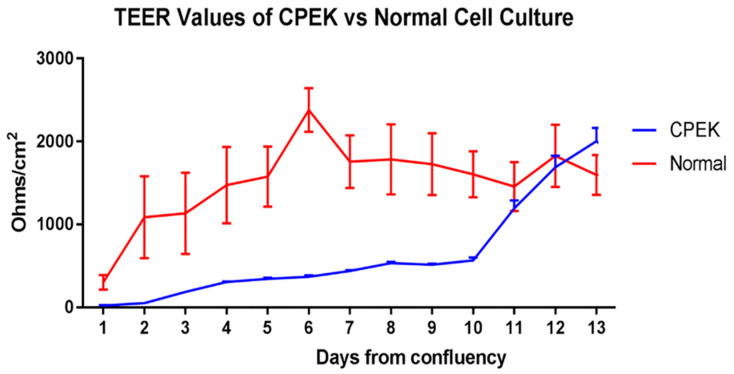
TransEpithelial Electrical Resistance (TEER) of canine progenitor epidermal keratinocyte (CPEK) cells (*n* = 3) and normal keratinocytes, NK (*n* = 4) in culture over a 13-day period. Graphs shows the mean and Standard Error of the Mean (SEM). Two-way Repeated Measures ANOVA showed a significant effect of time (*p* < 0.0001), group (*p* = 0.0473) and group x time interaction (*p* < 0.0001). Sidak’s multiple comparisons test showed a significant difference between CPEK and normal values on day 6 (*p* = 0.0002) and day 7 (*p* = 0.0406).

For TEER, ANOVA showed a significant effect of time (*p* < 0.0001), group (*p* < 0.0001) and group × time interaction (*p* < 0.0001) (Figure 2). CPEKs had lower TEER for the first 10 days and then drastically increased to reach similar values of NK. Variation on each time point is shown in standard error of the mean (SEM).

### 3.2. Assessment of Cell Morphology, Size, and Uniformity

**Figure 3:**
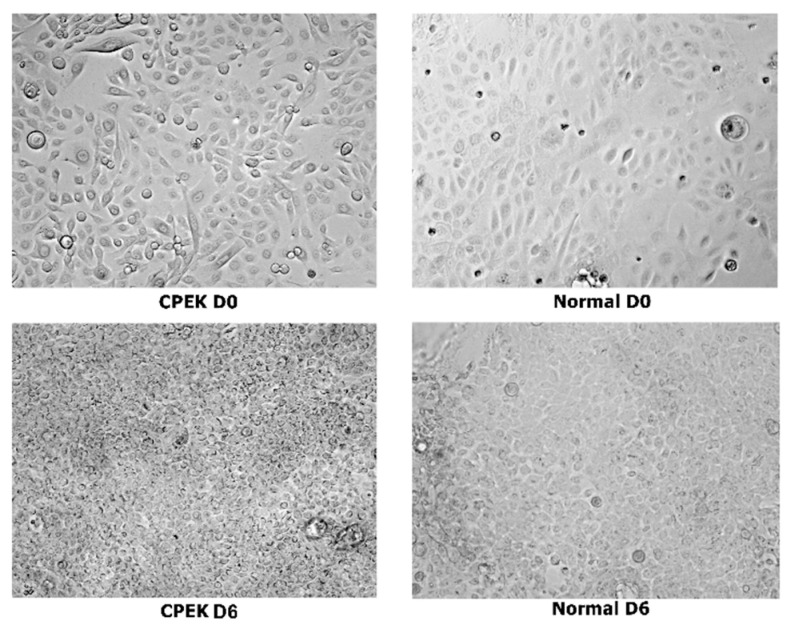
Composite showing unstained canine progenitor epidermal keratinocyte (CPEK) cells versus keratinocytes harvested from biopsies of normal dog at day 0 and day 6 of culture. CPEKs appear smaller and more numerous as time is progressing compared to NK. Images were taken at 40x.

**Figure 4:**
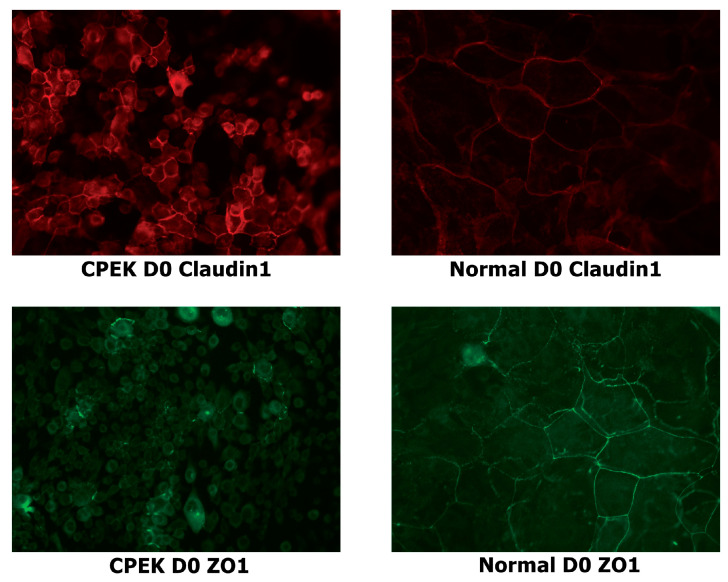
Stained canine progenitor epidermal keratinocyte (CPEK) cells and normal keratinocytes at day 0 of confluence. Normal keratinocytes have larger size and variation in size while CPEKs are visibly smaller. Claudin-1 staining is more intense in CPEK than Normal. ZO-1 immunofluorescent staining is cytoplasmic in CPEKs while is primarily on the cytoplasmic membrane in normal keratinocytes. Images were taken at 40x.

Images of CPEKs and keratinocytes harvested from normal dog’s skin biopsies are presented in Figure 3 and Figure 4.

**Figure 5:**
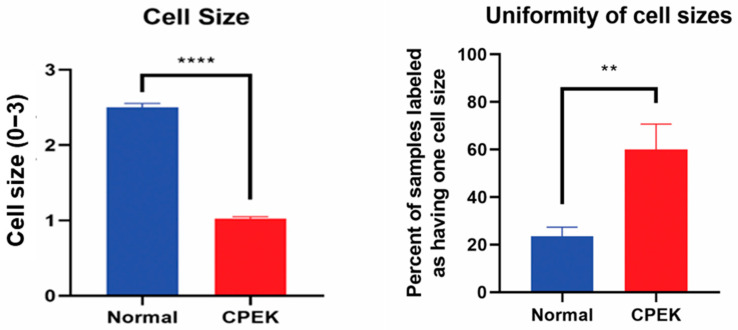
On the left, cell sizes in normal primary keratinocytes (Normal) vs. canine progenitor epidermal keratinocyte (CPEK) cells. Cell size was scored for size (1 = small, 2 = medium and 3 = large). Each observer evaluated 40 images for size and uniformity in a randomized fashion and being blinded to the source of the images. Observers labeled CPEK cells as having smaller cell sizes than normal. CPEK cell were shown to be significantly smaller using welch unequal variance *t*-test with a *p*-value of <0.0001. On the right, graph showing the variability of cell sizes between normal vs. canine progenitor epidermal keratinocyte (CPEK) cells. CPEK cells were shown to have a more uniform cell size across the sample (Observers labeled 60% of CPEK samples to have only once cell size present), while normal cells had a higher variability of cell sizes (Observers labeled 23.5% of normal cells to only have one cell size present). This difference was significant with a *p* value of 0.0078 using welch unequal variance *t*-test. **** indicates a significance *p* less than <0.0001 while ** indicates a significance of *p* higher than 0.001.

Size of CPEKs was significantly (*p* < 0.0001) smaller than primary NK and less variability of size was detected in CPEK (*p* = 0.0078) (Figure 5). Investigators scored that 60% of images of CPEKs had cells of one size while normal cells had a higher variability of cell sizes, only 20% showing one cell size. When cell size was scored from 1–3 (1 = small; 2 medium; 3 = large), all images of CPEKs were scored as 1 while normal keratinocytes had an average score of 2.5.

### 3.3. Tight Junction (TJ) Proteins

**Figure 6:**
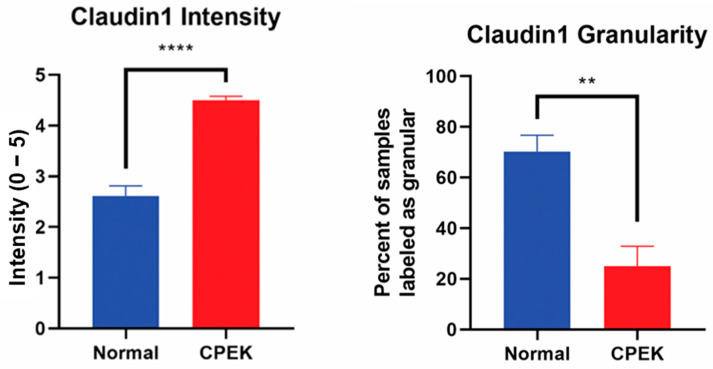
On the left, mean scores of the intensity of claudin-1 staining. Observers rated intensity from 0–5. Observer scores were averaged for each picture. Normal vs. canine progenitor epidermal keratinocyte (CPEK) cells scores were shown to be significantly different using welch unequal variance *t*-test with a *p*-value of <0.0001. On the right side, see the percent of samples labeled “granular” by the observers for the claudin staining. Observers were asked to labeled staining either granular or smooth. Normal vs. canine progenitor epidermal keratinocyte (CPEK) cells scores were shown to be significantly different using welch unequal variance *t*-test with a *p*-value of 0.0012. **** indicates a significance *p* less than <0.0001 while ** indicates a significance of *p* higher than 0.001.

**Figure 7:**
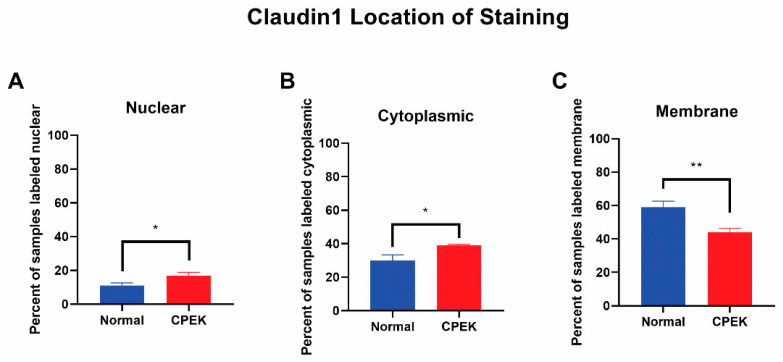
Claudin-1 location of staining. Percent shown is the number of observers who labeled staining in each location. Statistical analysis by welch unequal variance *t*-test showed normal vs. CPEK staining was significantly different in nuclear, cytoplasmic, and membrane staining with *p*-values of 0.0318, 0.0172, and 0.0026, respectively. * indicates a significance of *p* higher than 0.01 while ** is a significance of *p* less than 0.01.

For claudin-1 intensity of staining was greater in CPEKs (*p* < 0.0001) while granularity was less in CPEKs (*p* = 0.0012) (Figure 6). Claudin-1 was scored most of the time as membrane staining although some nuclear and cytoplasmic staining was reported. Claudin-1 nuclear and cytoplasmic staining greater in CPEK than normal (*p* = 0.0318 and *p* = 0.0172, respectively), and membrane staining was greater in normal than CPEK (*p* = 0.0026) (Figure 7). The continuousness of claudin-1 staining was not significantly different between normal and CPEKs.

**Figure 8:**
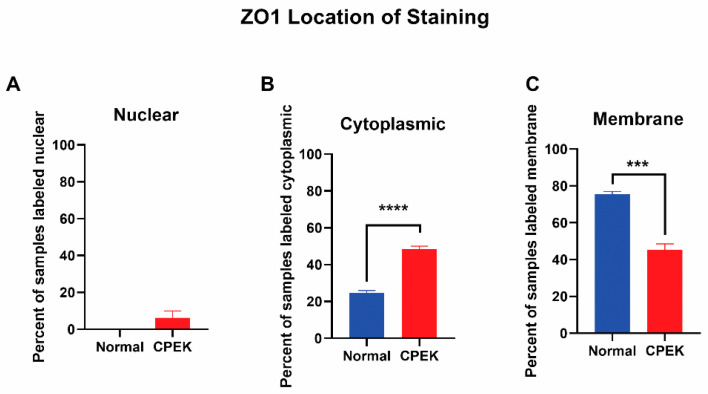
The location of ZO-1 staining. Percent shown is the number of observers who labeled staining in each location. (**A**) Nuclear staining was not significantly different between normal and CPEK, though normal cells had 0% of samples labeled as nuclear while CPEK had 6.19% labeled as nuclear. (**B**) Statistical analysis by Welch unequal variance *t*-test showed normal vs. CPEK cytoplasmic staining was significantly different (*p* < 0.0001), with CPEK cells having more cytoplasmic staining. (**C**) Normal vs. CPEK membrane staining was significantly different (*p* = 0.0002), with normal cells having more membrane staining. **** indicates a significance of less than 0.0001 while *** indicates a significance higher than 0.0001.

**Figure 9:**
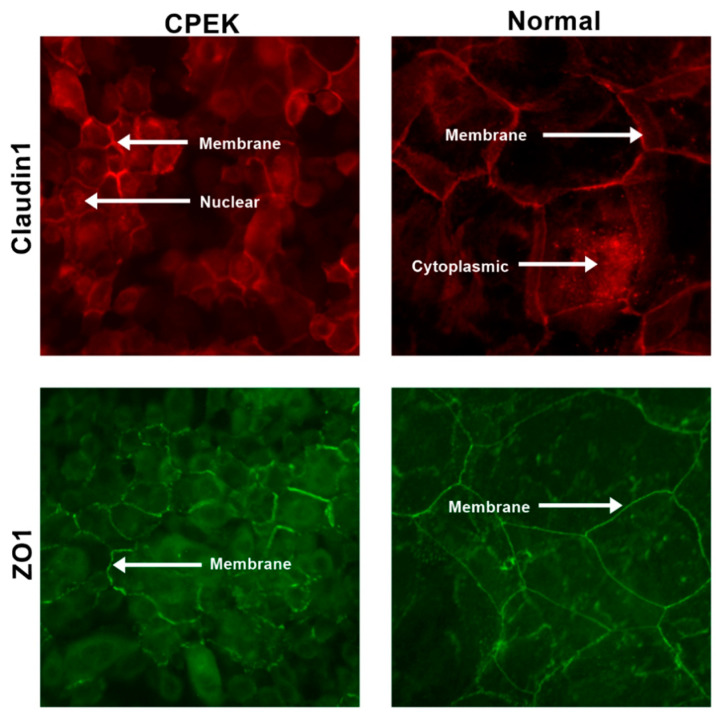
Representative images to show the location of the staining for both claudin and ZO-1 in both canine progenitor epidermal keratinocyte (CPEK) cells and normal keratinocytes. Staining is in red for claudin and green for ZO-1. Images were taken at 40x.

For ZO-1 staining, intensity and granularity were not significantly different between normal and CPEK cells. ZO-1 staining was mostly found in the membrane. Cytoplasmic staining of ZO-1 was higher in CPEKs than NK (*p* < 0.0001) and membrane staining was higher in normal than CPEK (*p* = 0.0002) (Figure 8). In addition, normal cells had staining that was more continuous than CPEK (*p* = 0.0002). Representative images of the location of the staining for TJ proteins is shown in Figure 9.

## 4. Discussion

In our study, CPEKs grown in monolayer did not perform similarly to the cultures of keratinocytes harvested from normal dogs in terms of epithelial permeability measurements like TEER. We also found differences in size and uniformity of CPEKs ad well as location and intensity of the staining for the TJ protein claudin-1. CPEKs also looked morphologically different from NK as they lacked the diversity of size and overall had a smaller size. Staining characteristics for claudin-1 and ZO-1 in CPEKs were different from NK in our study. We only evaluated ZO-1 and claudin-1 so more TJ proteins definitively should be investigated to increase our knowledge and understanding about similarities and differences in expression between CPEKs and NK. However, at this point in time, we can say that the location and intensity of TJ staining as well as TEER performance was not equivalent between CPEKs and normal canine keratinocytes grown in culture.

In the human literature several studies have reported on the used of primary normal human keratinocytes (NHEK) and TEER is measured [21,22]. Primary normal human keratinocytes are available from a single or pooled donors and may come from different body locations. It is interesting that TEER values measured on NHEK grown in monolayer is similar or even a little lower than what we found with CPEKs in our study when measured for up to 6 days [21]. Culture of immortalized human keratinocytes (HaCaT) has also been used in human medicine as model of the epidermis barrier and TEER has been measured over a course of 20 days [23]. No direct comparison was done with TEER of normal keratinocytes but it was reported that TEER of immortalized varied according to culture conditions [22] The authors reported that dynamic perfusion of culture media significantly improved the TJ formation as evidenced by measuring higher values of TEER compared to static culture [22]. This setting also maintained the high viability of cells over extended periods of time up to 17 days. In our study, the same culture conditions were used for CPEKs and primary keratinocytes and we did not do the dynamic perfusion of culture media.

More studies should be done in veterinary medicine to evaluate various culture conditions and how these conditions can be optimized to better replicate the behavior of normal keratinocytes so that CPEKs may still be used for permeability studies in the future. Ultimately the value of a vitro model is to assess changes when the system is exposed to various conditions. In our pilot studies, we did not assess the responsiveness to specific stimulations and only aimed at an initial assessment of CPEK performance as monolayer and the TEER measured compared to a monolayer of primary normal canine keratinocytes. This was different from what reported in a study in which CPEKs were grown to create a 3D epidermal model [14]. In such study it appeared that CPEKs could have the potential of being used as in vitro research tool to investigate TJ as the 3D model stained for claudin 1 and ZO-1.

Although CPEKs have been extensively used in veterinary medicine, no other publication about TEER using these cells was found in the literature. Published studies using CPEKS have focused on evaluating of cytokine production and reactivity of a variety of stimulants [24,25,26] and used them as representative of behavior of canine keratinocytes.

Our study has several limitations. One is the small number of normal dogs used for the biopsies and harvesting of the keratinocytes. Also, the assessment of tight junction was subjective based on images. Although three different investigators scored the images, this was not an objective measurement of protein or gene expression as it could have been done by western blot or PCR. Nevertheless, the images were quite consistently different among the two groups and provided some insight in the morphological differences between CPEKs and normal keratinocytes. Additional studies were done in our laboratory purchasing CPEKs to assess if the number of passages could affect the morphology and behavior of these cells and we found that this was not the case. Thus, we concluded that the morphological characteristics of CPEKs reported in this study were typical of this cells line.

## 5. Conclusions

We conclude that CPEKs grown in monolayer (2D) are not representative of NK for permeability studies. Morphological differences and differences in the staining of claudin and ZO-1 were also found between CPEKs and NK. Future studies should compare responsiveness to various stimulations between CPEKs and NK and how permeability characteristics would be changed.

## Data Availability

Data will be available upon request.
